# Effectiveness of Neurological Physiotherapy Interventions in Post-stroke Motor Rehabilitation: A Systematic Review

**DOI:** 10.7759/cureus.113639

**Published:** 2026-07-30

**Authors:** Siddareddy Ankireddypalli, Deepa Avadhani, Madhumita Singha Roy, Yogesh Kumar, Tejas Thakorbhai Prajapati, Neha Vyas, Rakesh Sahebrao Jadhav

**Affiliations:** 1 Department of Neurosurgery, Agartala Government Medical College and Govind Ballabh Pant Hospital, Agartala, IND; 2 Department of Neurology, Iswarya Hospital, Chennai, IND; 3 Department of Physical Medicine and Rehabilitation, Christian Medical College and Hospital, Ludhiana, IND; 4 Department of Physiology, All India Institute of Medical Sciences, Patna, IND; 5 Department of Physiology, Dr. N.D. Desai Faculty of Medical Science and Research Centre, Dharamsinh Desai University, Nadiad, IND; 6 Department of Physiotherapy, University of Engineering and Management, Jaipur, IND; 7 Department of Musculoskeletal Physiotherapy, Dr Bhanudas Dere College of Physiotherapy, Sangamner, IND

**Keywords:** brain-computer interface, motor rehabilitation, neurophysiotherapy, stroke, virtual reality

## Abstract

Stroke frequently results in persistent upper-limb, lower-limb, and gait impairments that restrict independence and quality of life. Neurological physiotherapy aims to enhance post-stroke motor recovery through task-specific practice, strengthening, sensory-motor retraining, feedback, neuroplasticity stimulation, and activity-based rehabilitation. This systematic review aimed to evaluate the effectiveness of neurological physiotherapy interventions in post-stroke motor rehabilitation and to summarize intervention characteristics, outcomes, methodological quality, and risk of bias. A systematic search was conducted across electronic databases, including PubMed, Google Scholar, ScienceDirect, and the Cochrane Library, to identify randomized controlled trials (RCTs) published from 2015 onward and available as full-text articles. After duplicate removal and screening, 47 full-text articles were assessed, and 11 RCTs were included. Data were extracted on study design, participants, stroke phase, intervention type, comparator, outcomes, numerical findings, and adverse events. Findings were synthesized narratively due to heterogeneity in intervention type, recovery phase, treatment dose, and outcome measures; no meta-analysis was performed. The review included functional strength training (FST), movement performance therapy, virtual reality (VR), robot-assisted rehabilitation, action observation therapy, mirror therapy, electrical stimulation, brain-computer interface (BCI)-assisted therapy, and walking-based interventions. Most interventions improved motor or functional outcomes, although superiority over conventional or dose-matched therapy was inconsistent. Overall, individualized, progressive, task-oriented neurological physiotherapy remains central to post-stroke motor recovery, with technology-assisted approaches offering additional value when integrated with meaningful functional practice.

## Introduction and background

Stroke is a major cause of long-term adult disability and often causes ongoing motor impairments that have a significant impact on independence, participation, and quality of life [1]. The most frequently observed motor deficits following stroke include weakness, abnormal muscle tone, deficits in motor coordination, decreased selective motor control, decreased sensory feedback, and decreased functional use of the affected limb [2]. Upper-limb impairment reduces the ability to reach, grasp, manipulate objects, dress, feed oneself, and attend to personal needs. At the same time, lower-limb dysfunction affects the ability to stand, walk without assistance, walk longer distances, walk without fatigue, and walk in community settings to perform activities of daily living (ADLs) [3]. Motor function is therefore a major focus of neurorehabilitation and its restoration. The goal of neurological physiotherapy is to maximize motor recovery through task-oriented practice, repetition, sensory-motor retraining, strengthening, postural control, gait training, feedback-based learning, and facilitation of neuroplastic adaptation [4]. Stroke characteristics, stroke phase, baseline motor severity, integrity of the corticospinal system, therapy intensity, and meaningful practice are all factors that affect post-stroke recovery [5]. The intensity of neurological plasticity during the early rehabilitation period can be leveraged, while the goal of chronic rehabilitation may be to enhance function, make better use of function, and prevent learned non-use [6]. The ability to select effective interventions necessitates evidence to establish the relationship between the physiotherapy intervention and motor and functional outcomes [7].

Current motor rehabilitation after stroke encompasses technology-supported and traditional methods. Functional strength training (FST) and movement performance therapy are still common methods for increasing the activity capacity of the upper limb [8]. Robot-assisted rehabilitation is used to support repetitive, intensive, and measurable upper-limb and gait recovery. Virtual reality (VR) offers enhanced multisensory environments, visual feedback, task progression, and engagement [9]. Visual-motor mechanisms are exploited in action observation therapy and mirror therapy for motor relearning. Functional electrical stimulation (FES) and brain-computer interface (BCI) therapy have been tried to synchronize motor intention and peripheral activation [10]. Walking capacity and real-world physical activity are addressed through high-intensity walking and step activity monitoring. Even with more interventions available, clinical interpretation is still challenging. Many trials show improvement within groups or phases compared with the same or similar doses of conventional therapy, but interventions are not always superior [11,12]. There is significant variation in the type of comparator design, feedback mode, therapist involvement, intensity, duration, and frequency of interventions. Outcome measures also vary across studies and include the Fugl-Meyer Assessment (FMA), Action Research Arm Test (ARAT), Box and Block Test (BBT), Functional Independence Measure (FIM), Barthel Index (BI), gait independence, 6-minute walk test (6MWT), walking speed, and daily step count. This heterogeneity makes direct comparison difficult and limits certainty regarding the optimal selection of interventions.

Another gap relates to the specificity of recovery. There are distinct differences among patients in the acute, subacute, and chronic phases of stroke in terms of their potential for spontaneous recovery, motor relearning ability, degree of impairment, and rehabilitation goals. Evidence from chronic stroke may not be directly applicable to early post-stroke rehabilitation, and short-term improvements may not translate into long-term functional outcomes. Adverse events are often incompletely reported, and several intervention trials are small, with few participants, pilot designs, and/or short follow-up periods. These concerns raise questions about the clinical utility, sustainability of benefits, and safety of these interventions in different patient populations. Another important gap is the difference between motor capacity and real-world performance. Changes in impairment measures or laboratory walking tests do not necessarily translate into greater use of the affected limb or increased daily walking activity. This is especially important in post-stroke rehabilitation, where functional independence is less about motor function and more about self-confidence, participation, environmental opportunities, and ongoing practice outside of therapy. Both impairment-level recovery and activity-level outcomes should be addressed in evidence synthesis.

A systematic review specifically focusing on randomized controlled trials (RCTs) of neurological physiotherapy interventions can help make sense of the evidence base for post-stroke motor rehabilitation. Organizing the findings by intervention type, stroke phase, target body region, outcome domain, and methodological quality makes it easier to interpret which methods have proven value and where uncertainty remains. This synthesis is needed to support clinical decision-making and identify future priorities for high-quality trials.

Objectives of the review

This review aimed to evaluate the effectiveness of neurological physiotherapy interventions in improving motor rehabilitation outcomes after stroke. It also aimed to summarize intervention characteristics, key motor and functional outcomes, methodological quality, and risk of bias across the included RCTs.

## Review

Methodology

Search Strategy

A systematic literature search was conducted using electronic databases, including PubMed, Scopus, Web of Science, PEDro, and the Cochrane Library, to identify primary research studies evaluating neurological physiotherapy interventions for post-stroke motor rehabilitation. The search focused on RCTs published from 2015 onward. Boolean operators ("AND" and "OR") were used to combine search terms related to the main concepts of stroke, motor rehabilitation, neurological physiotherapy, upper-limb recovery, lower-limb recovery, gait training, VR, robot-assisted therapy, FES, BCI, action observation therapy, mirror therapy, FST, and conventional physiotherapy. A representative search strategy was: (stroke OR "post-stroke" OR "cerebrovascular accident") AND ("motor rehabilitation" OR "motor recovery" OR "neurological physiotherapy") AND ("upper limb" OR "lower limb" OR gait OR "virtual reality" OR "robot-assisted therapy" OR "functional electrical stimulation" OR "brain-computer interface" OR "action observation therapy" OR "mirror therapy" OR "functional strength training" OR "conventional physiotherapy"). The initial database search identified 252 records. After removing 41 duplicates, 211 records were screened by title and abstract. Forty-seven full-text articles were assessed for eligibility, and 11 studies were included in the final review, as shown in the Preferred Reporting Items for Systematic Reviews and Meta-Analyses (PRISMA) flow diagram [13].

Eligibility Criteria

Studies were included if they were original primary research articles, RCTs, and involved adults with stroke receiving neurological physiotherapy or motor rehabilitation interventions. These interventions consisted of FST, movement performance therapy, VR rehabilitation, robot-assisted therapy, action observation therapy, mirror therapy, FES, BCI-assisted stimulation, gait training, high-intensity walking, and step-activity monitoring. Studies were required to report at least one motor, functional, gait, activity, impairment, or independence outcome from the original trial, including outcomes reported using previously published clinical scales, scores, indices, criteria, or standardized tests, such as the FMA, ARAT, BBT, FIM, BI/modified Barthel Index (MBI), Functional Ambulation Category (FAC), 10-meter walk test (10MWT), 6MWT, gait independence, walking speed, or daily step count. Studies published before 2015, protocols, editorials, conference abstracts, non-English publications, or studies lacking adequate outcome data were excluded. Systematic reviews, narrative reviews, scoping reviews, and meta-analyses were not incorporated into the main study findings or the results synthesis and were used only as background support in the Introduction and Discussion sections.

Data Extraction and Analysis

The data were extracted using a structured extraction sheet prepared according to the review objectives. Extracted information included author, year, country, study design, sample size, stroke phase, participant characteristics, intervention type, dose, duration, comparator, outcome measures, follow-up time points, statistical significance, adverse events, and study conclusions. Trial-level statistical parameters, including p-values, confidence intervals (CIs), mean changes, between-group differences, hazard ratios (HRs), chi-square values, effect sizes, responder counts, and clinically important response thresholds, were extracted as reported in the original RCTs and used only for narrative interpretation. The present systematic review did not directly administer any questionnaire, scale, score, criterion, index, or functional test to participants. Outcome tools such as the ARAT [14], Fugl-Meyer Assessment for the Upper Extremity (FMA-UE) [15], Wolf Motor Function Test (WMFT) [16], BBT [17], Chedoke-McMaster Arm and Hand Activity Inventory (CAHAI) [18], Stroke Impact Scale (SIS) [19], Modified Ashworth Scale (MAS) [20], Medical Research Council (MRC) muscle strength grading [21], surface electromyography (sEMG) [22], active range of motion (AROM) [23], FAC [24], 10MWT [25], 6MWT [26], FIM [27], and BI/MBI [28] were used only as reported in the included trials. No scoring sheets, manuals, item-level content, or proprietary materials were reproduced. Findings were synthesized qualitatively and narratively because of heterogeneity in intervention type, stroke phase, intensity, comparator design, follow-up duration, and outcome measures. No new inferential statistical testing, statistical pooling, meta-analysis, or meta-regression was performed. Accordingly, pooled effect estimates, forest plots, funnel plots, I², τ², and Cochran's Q statistics were not calculated. Heterogeneity was assessed qualitatively by comparing intervention modality, stroke recovery phase, comparator type, intervention dose, follow-up duration, and outcome measures across the included trials.

Quality Assessment

The methodological quality of the included studies was evaluated by analyzing the clarity and completeness of the study design, participant selection, description of the intervention, description of the comparator, reporting of outcomes, follow-up reporting, and interpretation of results. Studies with randomized designs, blinded outcome assessors, adequately described intervention protocols, standardized outcome assessment tools, and complete outcome data were considered to have higher methodological quality. Studies with small sample sizes, limited follow-up periods, limited reporting of allocation procedures, or pilot designs were interpreted more cautiously. The decision to exclude studies following final eligibility screening was not based on quality assessment criteria.

Risk of Bias Assessment

The Cochrane Risk of Bias 2 tool was used to evaluate the risk of bias in RCTs [29]. Sources of bias included in the assessment were deviations from intended interventions, outcome measurement, selection of reported results, missing outcome data, and bias arising from the randomization process. The overall judgment across these domains resulted in a rating of low risk of bias, some concerns, or high risk of bias for each study. This tool was chosen because all the included studies were randomized trials, it is widely used and readily available, and it is designed specifically for randomized trials. The most frequent concerns included limited blinding, small pilot samples, incomplete follow-up reporting, and performance bias due to rehabilitation interventions.

Results

Search Result

**Figure 1:**
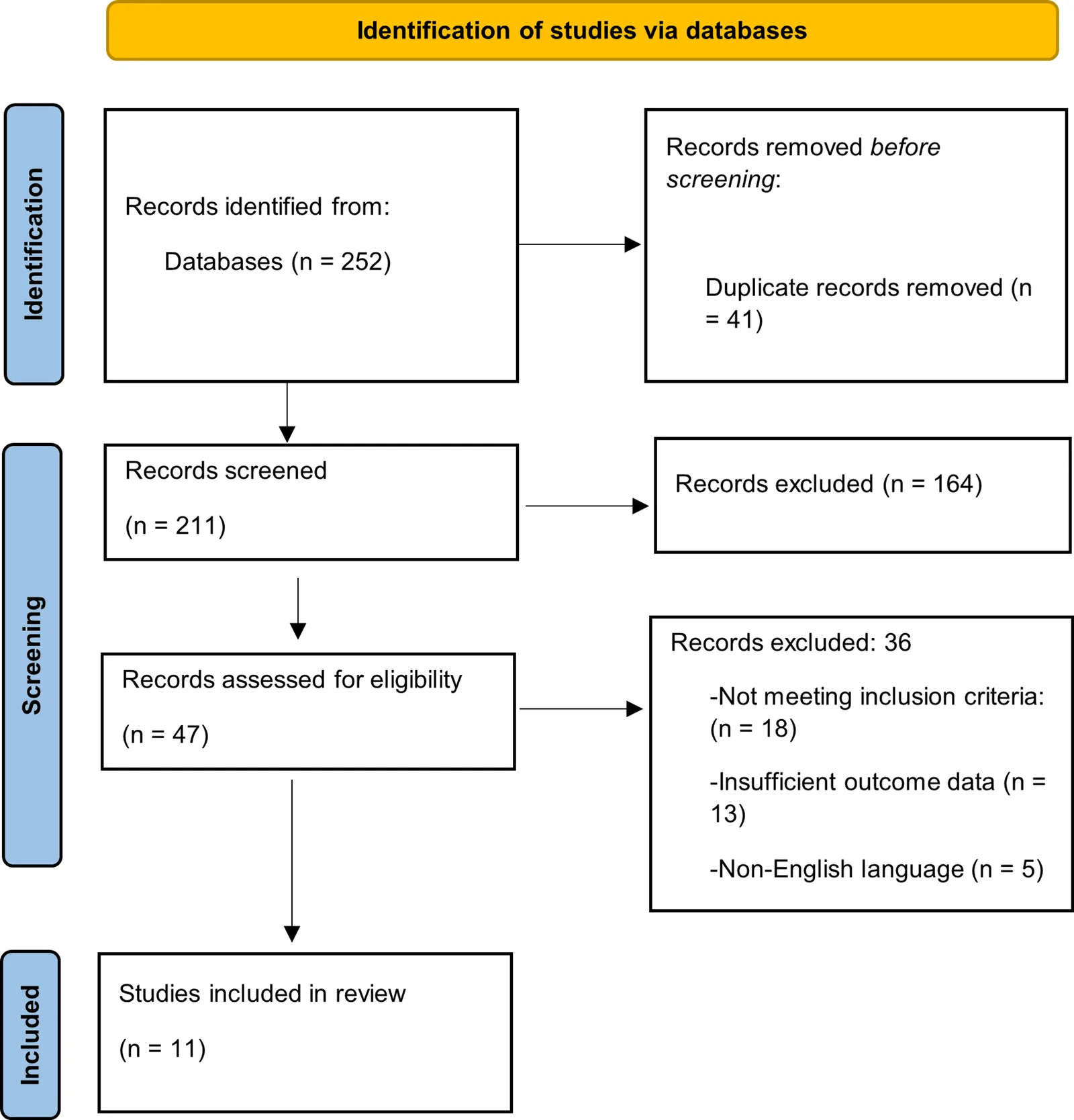
PRISMA flow diagram PRISMA, Preferred Reporting Items for Systematic Reviews and Meta-Analyses [13].

There were 252 records identified through the database search. After 41 duplicates were removed, 211 records remained for title and abstract screening. A total of 164 records were excluded during screening, leaving 47 full-text articles for eligibility assessment. Of these, 18 were excluded because they did not meet the inclusion criteria, 13 lacked sufficient outcome data, and five were not published in English. Eleven RCTs were included in the final review. The study selection process is presented in the PRISMA flow diagram (Figure 1).

Study Characteristics

The included trials were published between 2018 and 2024 and assessed the effectiveness of neurological physiotherapy approaches for motor rehabilitation after stroke. Sample sizes ranged from 21 to 288 participants, and intervention durations ranged from two to 12 weeks. Assessor-blinded randomized designs were used in most trials. Six studies primarily targeted upper-limb recovery, three targeted gait recovery or lower-limb rehabilitation, and two focused on general walking activity or functional outcomes. The interventions included VR, robot-assisted therapy, electrical stimulation, BCI-assisted therapy, action observation therapy, FST, and high-intensity walking. The Mini-Mental State Examination (MMSE) is a cognitive screening instrument used to assess cognitive status in clinical and research settings [30]. The key methodological characteristics, intervention protocols, outcome measures, and principal findings of the 11 included RCTs are summarized in Table 1.

**Table 1:** Characteristics and outcomes of included post-stroke neurophysiotherapy trials 10MWT: 10-meter walk test; 6MWT: 6-minute walk test; AOT: action observation therapy; ARAT: Action Research Arm Test; WMFT: Wolf Motor Function Test; AROM: active range of motion; BBT: Box and Block Test; BCI: brain-computer interface; BDNF: brain-derived neurotrophic factor; BI: Barthel Index; BoNT: botulinum toxin; CCFES: contralaterally controlled functional electrical stimulation; CI: confidence interval; COT: conventional occupational therapy; CPT: conventional physical therapy; DTI: Diffusion Tensor Imaging; EEG: electroencephalography; FAC: Functional Ambulation Category; FAS: full analysis set; SAM: step activity monitoring; FES: functional electrical stimulation; FIM: Functional Independence Measure; FMA: Fugl-Meyer Assessment; LE: lower extremity; UE: upper extremity; GRASP: Graded Repetitive Arm Supplementary Program; HR: hazard ratio; ICAM-1: intercellular adhesion molecule 1; IL-6: interleukin-6; MAS: Modified Ashworth Scale; MBI: modified Barthel Index; MCID: minimal clinically important difference; MMSE: Mini-Mental State Examination; MPT: movement performance therapy; MRC: Medical Research Council; NHPT: Nine-Hole Peg Test; NIHSS: National Institutes of Health Stroke Scale; NMES: Neuromuscular Electrical Stimulation; OT: occupational therapy; PPS: per-protocol set; PT: physical therapy; RCT: randomized controlled trial; ROM: range of motion; RPS: Reaching Performance Scale; sEMG: surface electromyography; SIAS: stroke impairment assessment set; SIS: Stroke Impact Scale; TMS: transcranial magnetic stimulation; UL: upper limb; VO₂: oxygen consumption; VR: virtual reality; VRT: virtual reality training; VRRS: virtual reality rehabilitation system; 8-OHdG: 8-hydroxy-2-deoxyguanosine; CAHAI: Chedoke-McMaster Arm and Hand Activity Inventory; FST: functional strength training; HTC: high tech computer corporation; HO-1: heme oxygenase; CONSORT: Consolidated Standards of Reporting Trials; TIDieR: Template for Intervention Description and Replication

Study	Design and setting	Participants and stroke phase	Intervention	Comparator	Outcomes and follow-up	Key findings	Safety/adverse events	Review-level interpretation
Hunter et al. [31]	Multicenter, observer-blinded RCT; three UK stroke centers. Allocation was concealed through an independent telephone randomization service; assessors were blinded where possible	n=288 adults, mean age, 72.2 years, recruited 2-60 days after anterior circulation stroke with residual upper-limb paresis and some voluntary contraction	FST+conventional physical therapy. Up to 90 min/day, 5 days/week, 6 weeks. Hands-off, progressive resisted strengthening embedded in functional task practice	Movement performance therapy+conventional physical therapy. Same intended dose. Hands-on facilitation, sensory input, mobilization, and quality-of-movement training	Primary: ARAT. Secondary: WMFT, grip force, pinch force. Neural measures: DTI/TMS variables. Outcomes after 6-week intervention and at 6 months post-stroke	ARAT improved in both groups, but the between-group difference was not significant. Mean ARAT change at outcome: FST+CPT, 9.70±11.72 vs. MPT+CPT, 7.90±9.18; p=0.298. At follow-up: 11.10 vs. 10.30; p=0.743. Neural measures did not correlate with or predict response	Pain and fatigue were monitored as adverse reactions. The main limitation was incomplete blinding at some outcome sessions because of staffing constraints	A large, methodologically strong RCT showing that two conceptually different upper-limb physiotherapy approaches produced similar recovery early after stroke. Supports task-specific strengthening as reasonable, but not clearly superior to movement-quality/facilitation therapy
Gandolfi et al. [32]	Single-blind, parallel-group RCT in outpatient neurorehabilitation settings	n=32 chronic stroke outpatients with upper-limb spastic hemiparesis; first-ever ischemic or haemorrhagic stroke; ≥6 months post-stroke; all received BoNT within the previous 12 weeks	Robot-assisted upper-limb training+botulinum toxin. Armotion end-effector robot; 10 sessions, 45 min/session, 2 sessions/week, 5 weeks. Included passive mobilisation/stretching plus game-based reaching/elbow tasks	Conventional upper-limb training+botulinum toxin. Same frequency and duration; passive mobilization/stretching plus scapular, shoulder, and elbow exercises	Primary: MAS. Secondary: FMA-LE, MRC strength scale. sEMG during the hand-to-mouth task in the experimental group. Pre/post only	No significant between-group difference for MAS or FMA. Robot group showed significantly greater improvement in overall upper-limb strength (p=0.004; d=0.49), shoulder abduction (p=0.039), external rotation (p=0.019), and elbow flexion (p=0.043)	No major safety concerns were reported in the retrieved text	Robot-assisted training was not superior for spasticity reduction or global FMA recovery, but may add value for strength gains when combined with BoNT in chronic spastic upper-limb paresis
Schuster-Amft et al. [33]	Multicenter, single-blind, parallel-group RCT in three Swiss rehabilitation outpatient departments	n=54 chronic stroke patients, mean age 61.3 years, ≥6 months post-stroke; moderate to severe arm/hand impairment; 22 allocated to VR and 32 to conventional therapy	VR-based upper-limb training using YouGrabber/Bi-Manu Trainer. 16 sessions × 45 min over 4 weeks. Real-time hand/finger/arm movement replication; modes included real movement, mirrored movement, and following movement; high-repetition grasp practice	Conventional physiotherapy/occupational therapy, same dose; task-related upper-limb treatment with neuromuscular, body-structural, perceptual, and sensory interventions	Primary: BBT. Secondary: CAHAI, SIS. Tested twice before, during, post-intervention, and at 2-month follow-up	Both groups improved. No significant between-group differences. BBT improved from baseline to follow-up in both groups; the authors reported similar effects, with most improvement in the first 2 weeks and persistence at 2 months. Fewer impaired patients appeared to benefit more from VR	Adverse events were registered; the study reported high compliance and very low dropout	VR-based training was not superior to dose-matched conventional therapy when delivered one-to-one, but may be useful to increase training repetitions and potentially extend therapy dose in group/home formats
Hernandez et al. [34]	Evaluator-blinded, 2-arm RCT with home-based intervention and 4-week follow-up	n=51 chronic stroke participants; treatment n=26, standard care n=25; first-time stroke >6 months earlier; mild to moderate upper-extremity impairment; no longer receiving rehabilitation services	Home-based VR serious game rehabilitation using Jintronix system with Kinect tracking; recommended 5 sessions/week, ≥20 min/session, 4 weeks. Therapist remotely monitored progress 1-2 times/week and adjusted task difficulty	GRASP home exercise program, standardized manual-based graded repetitive arm supplementary program; no remote therapist supervision	Primary: FMA-UE. Secondary: SIS, Motor Activity Log-14. Assessments at baseline, post-intervention, and 4-week follow-up. Feasibility metrics included installation, use time, pain, fatigue, dizziness, and falls	No statistically significant between-group differences. Overall time effect for FMA-UE (p=.045), especially baseline to post-intervention (p=.03). 9 VR participants reached MCID, compared with 3 standard-care participants; among active VR users, 9/16 achieved MCID, compared with 0/10 less-active users	Feasibility acceptable; home setup was described as simple and required limited space. Engagement was central to the response	Home VR was feasible and approximately comparable to evidence-based home exercise, with clinically meaningful gains concentrated among participants who actively engaged with the system
Huang et al. [35]	Assessor-blinded RCT with pre/post assessment	n=30 chronic stroke patients, randomized equally to VRT or COT; stroke onset >3 months; Brunnstrom stage >3; MMSE score >18 as reported by the original trial	Immersive VR-based motor-control training using HTC Vive; 16 sessions, 60 min/session, 2-3 days/week, supervised by an occupational therapist; 6-10 commercial VR tasks/session	Conventional occupational therapy with a pegboard, climbing ladder, stacking cones, and upper-limb training. Same session number/duration	Serum biomarkers: IL-6, ICAM-1, HO-1, 8-OHdG, and BDNF. Clinical outcomes: FMA-UE and AROM. Assessed pre- and post-intervention	Significant time effects for IL-6, HO-1, 8-OHdG, and most clinical outcomes. Significant group effects only for AROM elbow extension and forearm pronation. Significant time × group interactions for FMA-UE shoulder/elbow/forearm (p=0.004), FMA-UE total (p=0.008), and AROM shoulder flexion (p=0.001)	All participants completed the trial. Some reported eye strain, blurred vision, sweating, dizziness risk, and fatigue-related symptoms; no falls or accidents were reported in formal trial procedures	Immersive VR showed promising upper-limb motor benefits, particularly proximal FMA-UE and shoulder flexion, and introduced biomarker outcomes. Findings are limited by a small sample size and exploratory biomarker interpretation
Salvalaggio et al. [36]	Single-blind RCT in an inpatient stroke rehabilitation unit; CONSORT/TIDieR-informed reporting	n=124 stroke patients with upper-limb impairment; first unilateral cortical-subcortical stroke; 62 per group	VR rehabilitation with continuous “virtual teacher” feedback using VRRS. 1 h/day, 5 days/week, 4 weeks plus 1 h/day conventional therapy. The virtual teacher showed an ideal kinematic trajectory to emulate	Same VR rehabilitation without virtual teacher feedback, plus the same conventional therapy dose	Primary: FMA-UE. Secondary: FMA sensation, FMA pain/ROM, NHPT, BBT, RPS, MAS, FIM, and kinematic variables. Assessed pre- and post-intervention	No between-group advantage of virtual teacher. Responders by FMA-UE MCID: 36/62 teacher vs. 32/62 no-teacher; χ²=0.29, p=0.59. Both groups showed large within-group effects (Cohen’s d 1.14 and 0.92)	No prominent safety signal in retrieved text	Adding a continuous virtual-teacher display did not improve outcomes beyond standard multimodal VR feedback, but intensive combined VR+conventional therapy produced substantial within-group motor gains
Hsieh et al. [37]	Three-arm, single-blind pilot RCT with 3-month follow-up	n=21 subacute stroke patients; 1-6 months after unilateral ischemic or hemorrhagic stroke; FMA 20-60; age 20-80 years; 7 per group	Action observation therapy: first-person videos of AROM, reaching/object manipulation, and functional tasks, followed by physical execution. 15 sessions, 60 min/day, 5 days/week, 3 weeks, plus routine rehabilitation	Two comparators: mirror therapy and dose-matched bilateral arm training active control; both used the same movement/task categories and dose	Primary: FMA upper limb. Secondary: BBT, FIM, SIS. Baseline, post-treatment, and 3-month follow-up	FMA total mean change post-treatment: AOT 5.14, mirror therapy 2.57, active control 7.14. At 3 months: AOT 4.43, mirror therapy 4.71, active control 9.86. BBT post-treatment change: 6.14, 3.43, 5.14, respectively. AOT showed greater FIM improvement than other groups; mirror therapy generally had the least improvement	No adverse effects were reported	Pilot evidence suggests action observation therapy is at least comparable to active bilateral training and may outperform mirror therapy for functional independence, but the sample size was very small and not powered for definitive between-group inference
Huang et al. [38]	Parallel RCT; outcome assessors blinded	n=50 subacute stroke patients within 6 months post-stroke; first-ever stroke; Brunnstrom upper limb stage I-IV; 25 per group	CCFES to paretic wrist extensors, controlled by nonparetic wrist extension. Plus routine rehabilitation. 20 min/day, 5 days/week, 3 weeks	Cyclic NMES to paretic wrist extensors, same stimulation parameters/dose, plus routine rehabilitation	Primary: ARAT. Secondary: FMA-UE, BI, sEMG response of extensor carpi radialis and cocontraction ratio. Baseline and post-intervention	FMA-UE and BI improved in both groups. ARAT improved significantly only in the CCFES group. Between-group changes in ARAT, FMA-UE, and BI were not significantly greater with CCFES. sEMG response of paretic extensor carpi radialis improved more with CCFES (mean difference 0.09; 95% CI 0.01-0.16; p=0.026)	No adverse events during intervention or follow-up	CCFES may enhance paretic wrist-extensor activation more than NMES, but functional superiority was not demonstrated over a short 3-week dose
Luo [39]	Single-center, prospective RCT	n=64 acute ischemic stroke patients with lower-limb motor dysfunction; ≤2 weeks disease duration; Brunnstrom lower limb stage II-IV; NIHSS ≤15; 32 per group	Motor imagery-based BCI-controlled electrical stimulation+conventional rehabilitation. BCI delivered closed-loop EEG-triggered FES for foot dorsiflexion, foot inversion, knee extension, and calf external rotation. 1 h/day, 30 min morning+30 min afternoon, 2 weeks, 20 sessions, in addition to conventional rehabilitation	Conventional rehabilitation only: physical therapy 40 min+acupuncture 30 min daily, 5 times/week, 2 weeks, plus routine rehabilitation care	Primary: FMA-LE. Secondary: FAC, MBI. Pre/post after 20 sessions	Post-treatment outcomes favored BCI+conventional rehabilitation. FMA-LE: experimental 13.47±1.44 vs. control 10.97±1.60, p<0.001. FAC also favored intervention (p=0.031), and MBI improved more in the experimental group (p<0.001)	One participant reported fatigue, which resolved after rest; no other adverse events	Early closed-loop BCI-FES appears promising for acute lower-limb motor recovery and ambulation, but a single-center design and short follow-up limit certainty about durability
Hirano et al. [40]	Assessor-blinded, multicenter RCT in eight Japanese convalescent rehabilitation wards	n=91 first-ever hemiparetic subacute stroke patients unable to walk independently at baseline; age 20-80; onset within 60 days; FIM walk ≤3. FAS n=91; PPS n=85	Welwalk WW-1000 robot-assisted gait training+conventional physical therapy. Physical therapy 80 min/day, 7 days/week; Welwalk gait training 40 min/day, 6 days/week, 4 weeks as part of PT	Conventional physical therapy alone, 80 min/day, 7 days/week	Primary: gait independence, defined as FIM walk score ≥6, analysed as cumulative incidence over 25 weeks. Secondary: SIAS lower-limb motor/sensory/trunk verticality, FIM motor/cognitive, 10MWT, 6MWT, Wisconsin Gait Scale, gait pattern	No significant primary effect. At 25 weeks, gait independence in FAS: 23/45 Welwalk (51%) vs. 18/46 control (39%); cumulative incidence not significantly different (HR 1.523; 95% CI 0.813-2.853; p=0.189). The cerebral infarction subgroup showed a non-significant trend favoring Welwalk (HR 4.167; p=0.065)	No major safety issue was reported in the retrieved results	A well-designed multicentre trial found no statistically significant benefit of adding Welwalk to intensive conventional therapy, although subgroup trends suggest possible benefit in cerebral infarction requiring further testing
Thompson et al. [41]	Assessor-blinded, multisite RCT across four university/hospital laboratories; intention-to-treat analysis	n=250 chronic stroke participants, ≥6 months post-stroke; mean age ~63 years; walking without physical assistance; gait speed 0.3-1.0 m/s; <8000 steps/day. Groups: FAST-n=89, SAM-n=81, FAST+SAM n=80	Three interventions over 12 weeks, 2-3 sessions/week: FAST high-intensity walking at 70%-80% heart-rate reserve; SAM with individualized goals/coaching; FAST+SAM combined	Active comparison among the three intervention arms	Primary: steps/day. Secondary: 6MWT, self-selected walking speed, VO₂ at ventilatory threshold. Pre/post intervention	Steps/day increased significantly in SAM (+1542; 95% CI 1014-2069; p<0.001) and FAST+SAM (+1307; 95% CI 752-1861; p<0.001) but not FAST (+406; 95% CI −63 to 876; p=0.09). 6MWT improved in all groups: FAST +43 m, SAM +25 m, FAST+SAM +39 m. Walking speed improved in all groups; VO₂ did not change	No deaths or serious study-related adverse events; seven minor study-related adverse events and 11 serious non-study-related adverse events were reported	Behavioral step-monitoring with coaching was the key driver of increased real-world walking activity. FAST improved walking capacity but did not independently increase daily steps. FAST+SAM best addressed both walking capacity and walking performance

 Risk of Bias Assessment

The quality of the included randomized trials was generally good in terms of risk of bias. Random allocation and blinded outcome assessment were reported in most studies. Interventions in most trials were delivered through visible devices, therapist-led training, or behavioral coaching, making participant and therapist blinding difficult. Some concerns were mainly related to small sample sizes, incomplete follow-up reporting, limited allocation details, single-center design, and pilot or feasibility trial design. The methodological quality of larger multicenter trials was higher. The interpretation of intervention effects was therefore based on both the reported statistical significance parameters and the methodological limitations of each included trial. Overall, the risk profile suggests caution in interpreting intervention superiority, particularly in smaller pilot or feasibility trials. The overall risk of bias across the included randomized trials is summarized in Table 2.

**Table 2:** Risk of bias assessment of included studies

Study	Randomization	Allocation concealment	Assessor blinding	Incomplete outcome data	Selective reporting	Other bias	Overall judgement
Hunter et al. [31]	Low	Low	Some concerns	Some concerns	Low	Some concerns	Some concerns
Gandolfi et al. [32]	Low	Some concerns	Low	Low	Low	Some concerns	Some concerns
Schuster-Amft et al. [33]	Low	Low	Low	Low	Low	Some concerns	Low to moderate
Hernandez et al. [34]	Low	Low	Low	Some concerns	Low	Some concerns	Some concerns
Huang et al. [35]	Low	Some concerns	Low	Low	Low	Some concerns	Some concerns
Salvalaggio et al. [36]	Low	Low	Low	Low	Low	Some concerns	Low to moderate
Hsieh et al. [37]	Low	Low	Low	Some concerns	Some concerns	High	Moderate
Huang et al. [38]	Low	Low	Low	Low	Low	Some concerns	Low to moderate
Luo [39]	Low	Low	Some concerns	Low	Low	Some concerns	Some concerns
Hirano et al. [40]	Low	Low	Low	Low	Low	Some concerns	Low to moderate
Thompson et al. [41]	Low	Low	Low	Low	Low	Low	Low

Upper-Limb Motor Rehabilitation Outcomes

Eight trials were included in the assessment of upper-limb recovery using the ARAT, FMA-UE, BBT, MAS, MRC, FIM, SIS, and sEMG. FST, robot-assisted therapy, VR, action observation therapy, mirror therapy, and electrical stimulation were the interventions evaluated. Most studies reported within-group improvements, but between-group differences were inconsistent. Both FST and movement performance therapy were effective and produced similar ARAT changes. Improvements in selected strength measures and/or FMA-UE domains were observed following robot-assisted and immersive VR interventions. Action observation therapy was more effective than mirror therapy for several descriptive outcomes. CCFES had a greater effect on muscle activation than NMES. Upper-limb motor recovery outcomes across the included trials are summarized in Table 3, showing that most interventions produced within-group motor improvements, although between-group superiority was inconsistent.

**Table 3:** Upper-limb motor recovery outcomes across included trials ARAT: Action Research Arm Test; BBT: Box and Block Test; BoNT: botulinum toxin; CCFES: contralaterally controlled functional electrical stimulation; FMA: Fugl-Meyer Assessment; FMA-UE: Fugl-Meyer Assessment for Upper Extremity; GRASP: Graded Repetitive Arm Supplementary Program; MCID: minimal clinically important difference; MRC: Medical Research Council; NMES: Neuromuscular Electrical Stimulation; OT: occupational therapy; sEMG: surface electromyography; UL: upper limb; VR: virtual reality; FST: functional strength training

Study	Intervention	Comparator	Main outcome	Intervention value	Comparator value	Interpretation
Hunter et al. [31]	FST	Movement performance therapy	ARAT change	9.70	7.90	Comparable
Gandolfi et al. [32]	Robot-assisted UL training+BoNT	Conventional training+BoNT	MRC strength, p-value	0.004	-	Favored robot-assisted training
Schuster-Amft et al. [33]	VR upper-limb training	Conventional therapy	BBT follow-up mean	24.1	24.1 overall	Comparable
Hernandez et al. [34]	Home VR rehabilitation	GRASP home program	FMA-UE MCID responders	9	3	Favored active VR engagement
Huang et al. [35]	Immersive VR training	Conventional OT	FMA-UE interaction, p-value	0.008	-	Favored immersive VR
Salvalaggio et al. [36]	VR with a virtual teacher	VR without a virtual teacher	FMA-UE responders	36	32	Comparable
Hsieh et al. [37]	Action observation therapy	Mirror therapy	FMA change	5.14	2.57	Favored action observation
Huang et al. [38]	CCFES	NMES	sEMG mean difference	0.09	-	Favored CCFES

Lower-Limb Function and Gait Rehabilitation Outcomes

**Figure 2:**
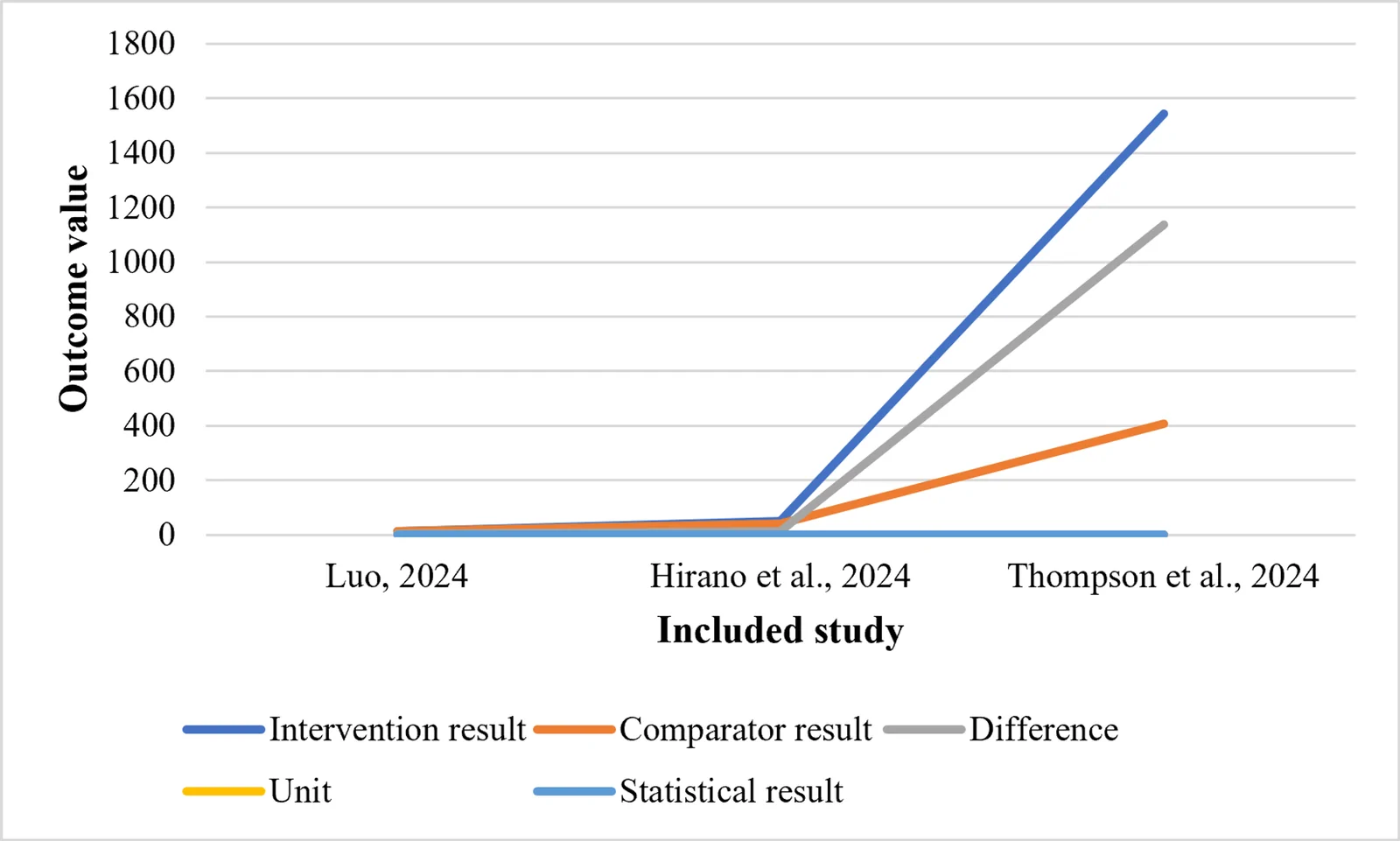
Lower-limb and gait rehabilitation outcomes across included trials Data are derived from Luo [39], Hirano et al. [40], and Thompson et al. [41].

Lower-limb and gait outcomes were reported in three trials that compared BCI-controlled stimulation, robot-assisted gait training, high-intensity walking, and step-activity monitoring. Conventional rehabilitation was less effective than BCI-controlled electrical stimulation for short-term improvements in FMA-LE, FAC, and MBI. Welwalk gait training assisted by the Welwalk robot demonstrated a greater numerical proportion of gait independence, although this difference was not statistically significant. Step-activity monitoring with coaching resulted in the greatest increases in real-world walking activity, whereas high-intensity walking primarily increased walking capacity, as measured by the 6MWT and walking speed. Lower-limb motor recovery, gait independence, and walking activity outcomes are summarized in Figure 2.

Intervention Modality and Recovery-Phase Distribution

The clinical heterogeneity of the evidence base included interventions across VR, robotics, electrical stimulation, BCI-assisted rehabilitation, task-specific physiotherapy, action observation, mirror therapy, and walking activity training. The most common modality was VR, and the largest number of participants received functional/task-specific physiotherapy and walking activity interventions. The most common recovery phase was chronic stroke, followed by early/subacute stroke. This distribution suggests that the review comprises both impairment-oriented recovery trials and activity-performance trials and requires interpretation by stroke stage and intervention type. The distribution of included studies by intervention modality, participant numbers, stroke recovery phase, and primary outcome domain is summarized in Table 4.

**Table 4:** Distribution of included studies by intervention type and stroke recovery phase VR: virtual reality; BCI: brain-computer interface; FMA-LE: Fugl-Meyer Assessment for Lower Extremity; FMA-UE: Fugl-Meyer Assessment for Upper Extremity; ADL: activity of daily living

Intervention category	Studies, n	Participants, n	The main recovery phase is represented	Main outcome domain
VR rehabilitation	4	259	Chronic/mixed	Upper-limb motor function
Robot-assisted rehabilitation	2	123	Chronic/subacute	Strength and gait independence
Electrical stimulation/BCI-assisted rehabilitation	2	114	Acute/subacute	FMA-UE, FMA-LE, muscle activation
Functional/task-specific physiotherapy	1	288	Early/subacute	Upper-limb activity capacity
Action observation/mirror therapy	1	21	Subacute	Upper-limb function and ADL
Walking activity/high-intensity gait training	1	250	Chronic	Steps/day and walking capacity

Discussion

This systematic review aimed to identify and synthesize evidence on neurological physiotherapy interventions in motor rehabilitation following stroke from 11 RCTs. The trials included diverse methods, such as FST, movement performance therapy, VR, robot-assisted rehabilitation, action observation therapy, mirror therapy, FES, BCI-assisted stimulation, high-intensity walking, and behavioral step-activity monitoring. In general, the results suggest that neurological physiotherapy interventions have some effect on improving motor outcomes after stroke, although this effect is inconsistent across different types of interventions and stages of recovery.

The largest number of trials focused on upper-limb rehabilitation. Across these studies, the ARAT, FMA-UE, BBT, MRC strength scale, FIM, and sEMG outcomes improved. Both FST and movement performance therapy showed similar benefits for early upper-limb motor recovery, indicating that the two interventions can be effective at equivalent treatment intensity. Selected improvements in strength were greater in the robotic intervention group, but robot-assisted upper-limb training did not clearly demonstrate superiority in motor outcomes or spasticity reduction. Based on these findings, robotic devices appear to enhance repetitive practice and muscle activation rather than provide greater overall functional improvements when task transfer is not adequately incorporated.

There were mixed results for VR interventions. Active use of VR systems showed gains in upper-limb motor function, engagement, and feasibility, with clinically meaningful improvements reported in several VR trials. Immersive VR also demonstrated positive results for FMA-UE domains and active ROM. However, for the majority of VR interventions, results did not consistently exceed those of traditional therapy at matched treatment doses. Considering this pattern, VR may be used as an additional therapeutic approach that can improve feedback, increase task repetition, and enhance access to home-based practice, but it is not consistently superior to therapist-led rehabilitation [8]. User engagement, impairment severity, task specificity, VR dose, and therapist-guided progression seem to influence the impact of VR [42].

Action observation therapy yielded superior improvements in some upper-limb outcomes, especially functional independence, compared with mirror therapy [43]. The observed benefit might be associated with the direct link between visual action observation and active execution; thus, motor planning and motor relearning could be facilitated by action-perception mechanisms [12]. The results of the included pilot trial were less consistent and suggest that the effect of visual illusion without active bilateral training or action observation may not be sufficient to achieve larger functional gains. This evidence should be interpreted with caution because of the small number of participants involved and should be confirmed with appropriately sized trials.

Electrical stimulation and BCI-assisted interventions demonstrated differences between physiological and functional outcomes. CCFES was more effective than NMES in activating paretic wrist extensors, but there was no apparent functional superiority after the brief intervention period studied. This indicates that more time might be needed to improve neuromuscular activation, more intense task integration, or a multi-functional approach to task training to achieve measurable gains in activity. In contrast, electrical stimulation using a motor imagery-based BCI in acute stroke showed significant effects on lower-limb motor function, FAC, and ADL. Closed-loop integration of motor intention and peripheral stimulation could provide a neuroplastic benefit, especially during early recovery when responsiveness to rehabilitation may be greater [44].

However, lower-limb and gait rehabilitation findings revealed a difference between walking capacity and actual walking in the real world. There was no statistically significant difference between the rate of gait independence achieved by conventional physical therapy and that achieved by Welwalk robot-assisted gait training, although the numerical proportion was higher in the latter. This finding indicates that the robot-assisted training program does not appear to be superior to intensive conventional gait training of similar duration, although certain subgroups may benefit. Step-activity monitoring with behavioral coaching had a significant effect on increasing daily step counts compared with high-intensity walking alone [45]. High-intensity walking has been shown to increase walking capacity, whereas step-activity monitoring with behavioral coaching was more effective in modifying walking behavior. This finding is clinically relevant because being able to walk in supervised environments does not necessarily mean that individuals will be able to move around independently in the community [46].

Direct comparisons are limited due to heterogeneity among the trials. There were variations in stroke phase at study entry, baseline impairment, intervention dose, setting, therapist involvement, device characteristics, comparator intensity, and outcome measures. There were more chronic stroke trials investigating VR and walking activity interventions, and more acute and subacute trials investigating BCI, electrical stimulation, action observation, and robotic gait training. The phase of stroke recovery is an important factor to consider because spontaneous recovery, neuroplasticity potential, compensatory learning, and functional goals vary over time following stroke [47].

Taken together, the results suggest that neurological physiotherapy is a fundamental component of post-stroke motor rehabilitation and that technology-supported therapy provides greater opportunities for intensity, feedback, engagement, and remote delivery. At this time, there is no evidence to support a single best intervention for all stroke populations. Selection of interventions should be based on the target impairment, phase of recovery, functional goals, available resources, patient involvement, and the need for activity transfer. Future trials should include larger sample sizes, standardized outcome measures, longer follow-up periods, standardized reporting of adverse events, and designs that measure both impairment-level recovery and real-world functional performance.

Limitations and future recommendations

The main limitations of this review are the clinical and methodological diversity of the included trials. The trials differed in stroke phase, number of participants, intervention type, intervention intensity, control group design, follow-up duration, and outcome measures, making direct comparison of the results difficult. Some trials had small sample sizes or pilot designs, which reduced the certainty of the effect estimates. Participant and therapist blinding were rarely possible because of the nature of rehabilitation interventions. No meta-analysis was undertaken, and only narrative synthesis was used.

Future studies should use larger multicenter RCT designs with standardized outcome measures, longer follow-up periods, and clearer reporting of intervention dose, adherence, adverse events, and functional carryover. Participants should be stratified according to stroke phase, baseline impairment, and motor recovery potential. Future evidence needs to evaluate functional capacity as well as real-world use, such as arm use during the day, community walking, participation, and quality of life. Technology-assisted interventions should be evaluated as part of task-oriented rehabilitation rather than as stand-alone interventions.

## Conclusions

This systematic review shows a positive effect of neurological physiotherapy interventions for post-stroke motor rehabilitation; the evidence is strong for certain interventions, stroke phases, and outcome domains but less consistent for others. The RCTs included in this review demonstrated the effectiveness of functional strength training, movement performance therapy, VR, robot-assisted rehabilitation, action observation therapy, electrical stimulation, brain-computer interface-assisted therapy, and walking-based interventions for improving upper-limb function, lower-limb motor recovery, gait performance, or daily activity. No single intervention performed better than conventional or dose-matched rehabilitation for all patients, indicating that patient characteristics, baseline impairment, stage of recovery, intervention intensity, task specificity, and treatment engagement are important considerations for treatment effectiveness. Technology-assisted methods seem to have the greatest value when they augment or are combined with repetition, feedback, motivation, and access to functionally meaningful, therapist-guided practice. Well-structured, progressive, task-oriented rehabilitation and real-world step-activity monitoring strategies were best supported. Larger sample sizes, longer follow-up periods, standardized outcome measures, clearer reporting of adverse events, and functional measures of daily-life performance should be incorporated into future studies. Neurological physiotherapy remains relevant for optimizing motor recovery after stroke but should be delivered in an individualized manner.
